# Supplementary material for: Predicting visual function by interpreting a neuronal wiring diagram
Source: Nature. 2024 Oct 2;634(8032):113–23. doi: 10.1038/s41586-024-07953-5 (PMC11446822; doi:10.1038/s41586-024-07953-5)
Supplement: Supplementary file 6 — CRF and ERF predictions for individual TmY4 and TmY9 cells. Analogous to Supplementary Data 3, but for TmY target types. Shown are the top four monosynaptic pathways, the strongest pathway passing through each of the top ten intermediary types (ranking from Extended Data Fig. 7), and the trisynaptic pathway Tm1–TmY–Dm3–TmY (see the section entitled Prediction of spatial normalization). [file 41586_2024_7953_MOESM6_ESM.zip › DataS4/TmY9qΓèÑ/720575940627106916.pdf]

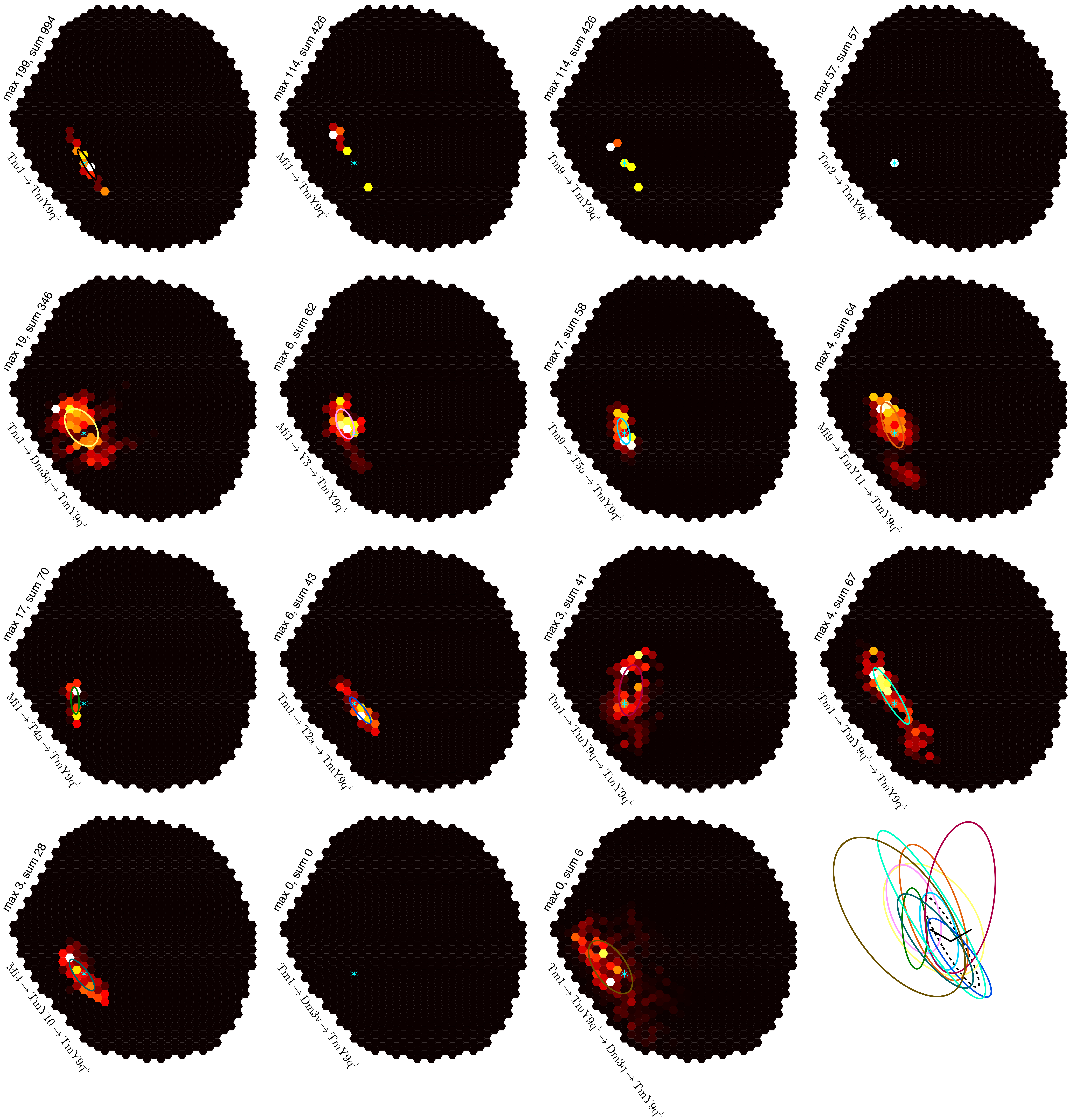

max 199, sum 994
Tm1 → TmY9q⊥
max 114, sum 426
Mi1 → TmY9q⊥
max 114, sum 426
Tm9 → TmY9q⊥
max 57, sum 57
Tm2 → TmY9q⊥
max 19, sum 346
Tm1 → Dm3q → TmY9q⊥
max 6, sum 62
Mi1 → Y3 → TmY9q⊥
max 7, sum 58
Tm9 → T5a → TmY9q⊥
max 4, sum 64
Mi9 → TmY11 → TmY9q⊥
max 17, sum 70
Mi1 → T4a → TmY9q⊥
max 6, sum 43
Tm1 → T2a → TmY9q⊥
max 3, sum 41
Tm1 → TmY9q → TmY9q⊥
max 4, sum 67
Tm1 → TmY9q⊥ → TmY9q⊥
max 3, sum 28
Mi4 → TmY10 → TmY9q⊥
max 0, sum 0
Tm1 → Dm3v → TmY9q⊥
max 0, sum 6
Tm1 → TmY9q⊥ → Dm3q → TmY9q⊥
